# Colorectal cancer screening practices and perceptions among the general population of the southwestern region of Saudi Arabia

**DOI:** 10.1097/MD.0000000000044461

**Published:** 2025-09-12

**Authors:** Syed Esam Mahmood, Mousa Ghazwani, Fatima Riaz, Ausaf Ahmad, Abdullah A. Alsabaani, Ayed A. Shati, Rishi Kumar Bharti, Razia Aftab Ahmed

**Affiliations:** a Department of Family and Community Medicine, College of Medicine, King Khalid University, Abha, Saudi Arabia; b Department of Pediatric Surgery, Maternity and Children Hospital, Abha, Aseer, Saudi Arabia; c Department of Community Medicine, Kalyan Singh Government Medical College Bulandshahr, Uttar Pradesh, India; d Department of Child Health, College of Medicine, King Khalid University, Abha, Saudi Arabia.

**Keywords:** colorectal cancer, perceptions, population, Saudi Arabia, screening

## Abstract

The 3rd most frequent cancer reported in the world for both sexes is colorectal cancer (CRC). Additionally, the frequency of CRC is rising in younger people, and by 2030, more people aged 20 to 49 years are anticipated to have the disease. This study was undertaken to assess the CRC screening practices and perceptions among southwestern region of Saudi Arabia and to suggest suitable preventive and promotive measures to the study population. This cross-sectional study included individuals who were 18 years of age and older, from the general population of the southwestern region. Three hundred forty participants were recruited using a convenience sampling technique. Data were collected using a self-administered questionnaire. A low screening rate of 4.7% was observed, with no significant differences by age, gender, marital status, or income. Employed individuals had slightly higher rates (5.0%) than the unemployed (3.4%). Those with a family history of CRC showed a higher screening rate (11.1%) compared to those without (3.9%), though this was not statistically significant. Awareness of CRC screening was low, with 58.8% unaware of any tests; among the informed, lower GI endoscopy was the most recognized method. While perceptions of CRC were generally positive, with many believing in its preventability and the importance of early detection, 80.0% had never considered screening, mainly citing cost as a barrier. The study underscores a significant gap in CRC screening uptake and awareness, highlighting the need for targeted educational interventions. There is a need for health education and health promotion activities for the prevention of CRC risk factors. The effective implementation of regular screening program for the early detection of the disease in the community is a necessity.

## 1. Introduction

The 3rd most common cancer and the 2nd most lethal, respectively, is colorectal cancer (CRC).^[1]^ Globally, a rise in CRC incidence has been seen during the previous 10 years. Additionally, the frequency of CRC is rising in younger people, and by 2030, more people aged 20 to 49 are anticipated to have the disease.^[2]^

The fact that death is much lower than incidence reflects the generally favorable prognosis for CRC.^[3]^

Nearly 1380 persons were diagnosed with colon cancer in 2013, which accounts for 11.9% of all cancer diagnoses, according to the Saudi Arabian cancer incidence report. In this group, 53.1% of men and 46.9% of women were afflicted by colon cancer in 2013.^[4]^

Colon cancer survival is directly correlated with the pathological and clinical stage at diagnosis, and various findings indicate that if CRC develops in childhood, it is connected to increased mortality and a more severe illness, which is especially pertinent to the Saudi population.^[5]^

Age has been identified as a primary determinant in the occurrence of CRC; after the age of 40, the risk of a diagnosis increases, and it increases significantly beyond the age of 50. Several other variables, including familial or hereditary polyposis, alcohol, smoking, and ulcerative colitis, have also been connected to a higher chance of developing CRC.^[6]^

The consumption of specific diets and the onset of CRC are strongly correlated, and the prevention of the disease may be possible by altering dietary practices.

The results of the literature review showed that screening program participation and late illness diagnosis were impacted by awareness of risk factors and the ability to recognize early signs. Because of this, participation in screening programs is favorably impacted by knowledge about the condition.^[7]^

Nevertheless, the incidence and mortality of CRC can be lowered by screening. There are many kinds of CRC screening tests, including invasive and noninvasive procedures like flexible sigmoidoscopy and colonoscopy. Noninvasive procedures include fecal occult blood testing (FOBT) and stool DNA testing. According to numerous research studies, screening can lower CRC mortality. For instance, fecal occult blood test, a straightforward noninvasive self-screening test that detects minute amounts of blood in stool, can cut CRC mortality by 43%, as well as flexible sigmoidoscopy which can provide similar results.^[8]^

In Saudi Arabia, the majority of CRC patients are diagnosed at an advanced stage with metastases, which makes therapy challenging and raises mortality. By being aware of the early signs of CRC, risk factors can be reduced. By identifying CRC at an early stage, screening can also help to lower CRC mortality.^[8]^

Therefore, it is of most importance that people have sufficient knowledge and understanding of CRC, they should be aware of its symptoms and signs. In addition, they should also know at what age or circumstance they should seek medical screening facilities to find any abnormalities and reduce their risk of CRC morbidity and mortality. In Saudi Arabia, the Ministry of Health has introduced a free early detection program for CRC aimed at reducing mortality through timely diagnosis and treatment referrals. This program is available to Saudi nationals and expatriates in government sectors at designated health centers.

Given the alarming rise in CRC incidence, particularly among younger populations under 50, and the challenges associated with late-stage diagnoses in Saudi Arabia, this study addresses a critical gap in understanding knowledge and practices regarding CRC screening among adults aged 18 and above in the Southwestern region. Prior research has highlighted low awareness and screening practices among older adults, underscoring the need for updated data that encompasses a broader age range. By including individuals under 40, this study aims to identify age-specific risk factors, promote earlier screening, and ultimately improve outcomes while reducing late-stage diagnoses. The unique lifestyle and environmental risks faced by younger populations necessitate their inclusion in CRC studies to inform evolving screening guidelines and policy decisions. Through assessing the perceptions and screening practices of the general population, this research seeks to inform health authorities and contribute to the development of targeted interventions and policies, which are essential for improving early detection rates, reducing mortality, and controlling the spread of CRC in the region.

## 2. Materials and methods

### 2.1. Study design and setting

In the Southwestern region, Aseer Region has an estimated population of 2.212 million, while Jazan region has a population of around 1.568 million. This analytical cross-sectional study was conducted in the general population of Southwestern region.

### 2.2. Inclusion and exclusion criteria

The study includes individuals who are 18 years of age and older, it includes both males and females. The study includes all Saudi residents in Southwestern region. All those within those 2 regions who participated according to the parameters given above and who gave consent were included in the study. Participants who were younger than 18 years and those who had CRC were excluded. The study also excludes those who were unable to write or read Arabic Language in the Arabic version of the questionnaire. Participants who were not able to read or write in English were excluded from the English version of the questionnaire. Also, those who did not give consent were exempted from the study.

### 2.3. Sampling technique

The participants were recruited using a convenience sampling technique. The collection of data was done using an online self-administered questionnaire by a team of principal author and coauthors.

### 2.4. Data collection

Data collection was conducted using an online self-administered questionnaire designed to gather information relevant to the study objectives. The questionnaire consisted of closed-ended questions to ensure clarity and facilitate quantitative analysis. It was developed in both English and Arabic using Google Forms, allowing for inclusivity and accessibility. To reach a broad audience, the questionnaire was distributed among the general population through social media platforms such as Twitter, WhatsApp, and Instagram, enhancing the convenience of data collection. This approach ensured a wide reach and allowed for the inclusion of diverse demographic groups.

The survey items were based on a comprehensive literature review^[9,10]^ and were further refined through revision by a panel of experts in the field. To validate the questionnaire’s effectiveness, it underwent a pilot test with 30 participants to evaluate its clarity and relevance. Feedback from the pilot participants led to adjustments in wording and question structure, thereby enhancing the questionnaire’s clarity and reducing ambiguity. The pilot study also assess reliability through Cronbach alpha, resulting in a coefficient of 0.85, indicating good internal consistency and reliability. To ensure accuracy and cultural relevance, the questionnaire was translated from Arabic to English by a bilingual expert, who carefully considered the context and nuances of each question. This rigorous translation process included back-translation, where the English version was translated back into Arabic by a different bilingual individual, to identify any discrepancies or loss of meaning. This 2-step process minimized potential bias by ensuring that participants from both language groups interpreted the questions consistently and understood them in their intended context. The questionnaire comprises several sections, including demographic information and queries regarding knowledge, attitudes, practices, and perceptions related to CRC. This comprehensive structure is designed to provide a holistic view of the participants’ insights and experiences concerning CRC.

Participants answered the questions with “yes,” “no,” and “I do not know.” If the participants answered a question correctly, they had a score of 1, and if they answered the questions incorrectly or “I do not know” they did not get a score (0).

The 3 attitude questions were answered using a Likert scale (strongly agree, agree, neutral, disagree, and strongly disagree).

BMI of participants were categorized as, underweight (<18.5 kg/m^2^), normal or lean BMI (18.5–22.9 kg/m^2^), overweight (23.0–24.9 kg/m^2^), and obese (≥25 kg/m^2^).^[11]^

### 2.5. Sample size

The sample size is calculated using the following formula:


$$\begin{array}{l} N=Z^{2}\times P\times \left(1-P\right)/C2 \end{array}$$

N = sample size, Z = 1.96 at 95% level of significance and 80% power *P* = prior prevalence of knowledge of screening tests (52%),^[12]^ and C = degree of precision (0.05). The margin of error is 5.32%. The estimated minimum sample size is 340.

### 2.6. Data analysis

Statistical analysis was done using the Statistical Package for the Social Sciences version 16.0 for Windows (SPSS Inc., Chicago). The chi-square test was used to compare differences in baseline characteristics and screened or tested for CRC. Qualitative variables were presented by descriptive statistics. Quantitative variables were grouped into categories (e.g., age ranges, income brackets) and expressed as frequency counts and percentages to facilitate analysis and interpretation. Statistical significance was set at the .05 level

### 2.7. Ethical approval and informed consent

The ethical approval of IRB was obtained from Deanship of Scientific Research (RGP2/189/45), King Khalid University on March 23, 2023 before starting to collect the data of participants.

Informed consent was secured from participants. Participation was voluntary. The questionnaire started with a brief explanation of its objective and intent and a reminder to participants that their participation is entirely voluntary. The survey did not collect names, or dates of birth or addresses. Electronic informed consent was obtained from all the participants before filling out the survey forms. The confidentiality of data was well-preserved throughout the study by keeping it anonymous and asking the participants to select honest answers and options. All collected data was only used for research purposes.

## 3. Results

This cross-sectional study included individuals who were 18 years of age and older, from the general population of the southwestern region. Three hundred forty participants were recruited using a convenience sampling technique. In which, Saudis were 336 (99%) and non-Saudis were 04 (01%).

### 3.1. Sociodemographic and risk factors

Table 1 presents the sociodemographic characteristics of respondents who have ever been screened or tested for CRC. The analysis of age groups reveals that among individuals <40 years old (n = 256), 4.7% have undergone CRC screening, while 95.3% have not. Similarly, for those aged 40 and above (n = 84), the screening rate is 4.8%, with 95.2% not having been screened. A higher percentage of males (6.3%) have been screened or tested for CRC compared to females (1.7%), though the difference is not statistically significant. Respondents with higher education levels (bachelor’s degree and postgraduate degree) show slightly higher rates of screening compared to those with secondary education. Employed individuals have a significantly higher rate of CRC screening compared to the unemployed. There is no significant difference in screening rates between married and single individuals. Respondents with a monthly income (Saudi Arabian Rials) of <5000 and those with income between 5000 to 9999 show relatively higher rates of screening compared to those with higher income brackets. Analysis indicates that employment status significantly influences CRC screening rates, while other factors such as age, gender, education, marital status, and income do not show statistically significant associations with screening behavior among the respondents.

**Table 1:** Sociodemographic characteristics of respondents ever been screened or tested for CRC.

Sociodemographic characteristics		Ever been screened or tested for CRC		Total (n = 340)	*P*-value	OR (95% CI)
		No (n = 324)	Yes (n = 16)			
Age	<40 yr	244	12	256	.978	1.02 (0.32–3.24)
		95.3%	4.7%	100.0%		
	40 yr and above	80	4	84		
		95.2%	4.8%	100.0%		
Gender	Female	116	2	118	.056	3.90 (0.87–17.48)
		98.3%	1.7%	100.0%		
	Male	208	14	222		
		93.7%	6.3%	100.0%		
Education	Secondary education	70	4	74	.073	Ref.
		94.6%	5.4%	100.0%		
	Bachelor’s degree	206	6	212		2.19 (0.55–8.67)
		97.2%	2.8%	100.0%		
	postgraduate degree	48	6	54		2.62 (0.58–11.74)
		88.9%	11.1%	100.0%		
Occupation	Employed	268	14	282	**.008**	1.46 (0.32–6.61)
		95.0%	5.0%	100.0%		
	Unemployed	56	2	58		
		96.6%	3.4%	100.0%		
Marital status	Married	242	12	254	.977	1.01 (0.31–3.23)
		95.3%	4.7%	100.0%		
	Single	82	4	86		
		95.3%	4.7%	100.0%		
Monthly Income (Saudi Arabian Riyals)	<5000	94	2	96	.070	Ref.
		97.9%	2.1%	100.0%		
	5000 to 9999	30	2	32		0.32 (0.71–1.47)
		93.8%	6.2%	100.0%		
	10,000 to 15,000	126	4	130		0.51 (0.06–4.32)
		96.9%	3.1%	100.0%		
	More than 15,000	74	8	82		022 (0.03–1.54)
		90.2%	9.8%	100.0%		

Bold value indicates statistically significant result at the .05 level.

CI = confidence interval, CRC = colorectal cancer, OR = odds ratio.

Table 2 presents an analysis of risk factors among respondents who have undergone CRC screening, categorizing individuals based on their smoking status, BMI, family history of CRC, and the specific relation of participants to those with a family history of CRC. Current smokers exhibit a 5.0% screening rate, while ex-smokers show a 0.0% screening rate, and nonsmokers have a 5.1% screening rate. Individuals with underweight, normal/lean BMI, overweight, and obese BMI have screening rates of 0.0%, 3.1%, 7.1%, and 5.2%, respectively. Those with a family history of CRC have an 11.1% screening rate, whereas those without a family history exhibit a 3.9% screening rate. The difference in screening rates approaches statistical significance, suggesting a potential association between family history and increased likelihood of screening. Participants with first-degree relatives with CRC show a 10.0% screening rate, second-degree relatives exhibit an 11.5% screening rate, and those with no family history have a 3.9% screening rate. Individuals with a family history of CRC may be more inclined to undergo screening, although this association is not conclusively significant. Smoking status and BMI, on the other hand, do not show a significant association with CRC screening behavior among the respondents.

**Table 2:** Risk factors among respondents ever been screened or tested for CRC.

Risk factors		Ever been screened or tested for CRC		Total (n = 340)	*P*-value	OR (95% CI)
		No (n = 324)	Yes (n = 16)			
Smoking Status	Current smoker	38	2	40	.470	Ref.
		95.0%	5.0%	100.0%		
	Ex-smoker	28	0	28		1.47 (0.12–14.07)
		100.0%	0.0%	100.0%		
	Nonsmoker	258	14	272		0.96 (0.21–4.43)
		94.9%	5.1%	100.0%		
BMI	Underweight (<18.5 kg/m^2^)	28	0	28	.459	Ref.
		100.0%	0.0%	100.0%		
	Normal or lean BMI (18.5–22.9 kg/m^2^)	62	2	64		0.43 (0.02–9.43)
		96.9%	3.1%	100.0%		
	Overweight (23.0–24.9 kg/m^2^)	52	4	56		0.20 (0.01–3.93)
		92.9%	7.1%	100.0%		
	Obese (>25 kg/m^2^)	182	10	192		0.30 (0.02–5.34)
		94.8%	5.2%	100.0%		
Family history of CRC	Yes	32	4	36	.055	3.04 (0.92–9.98)
		88.9%	11.1%	100.0%		
	No	292	12	304		
		96.1%	3.9%	100.0%		
Relation of participants those who have a family history of CRC	No family history	292	12	304	.198	Ref.
		96.1%	3.9%	100.0%		
	First-degree relatives	9	1	10		0.36 (0.04–3.15)
		90.0%	10.0%	100.0%		
	Second-degree relatives	23	3	26		0.31 (0.08–1.19)
		88.5	11.5%	100.0%		

CI = confidence interval, CRC = colorectal cancer, OR = odds ratio.

A statistically significant association between occupation status and the likelihood of having been screened or tested for CRC, with a *P*-value of .008. Among the employed respondents, 5.0% had been screened or tested for CRC, whereas among the unemployed respondents, only 3.4% had undergone screening or testing. This suggests that employment status may influence the likelihood of individuals getting screened for CRC, with employed individuals being slightly more likely to have been screened compared to unemployed individuals.

### 3.2. Awareness and knowledge

**Figure 1.**
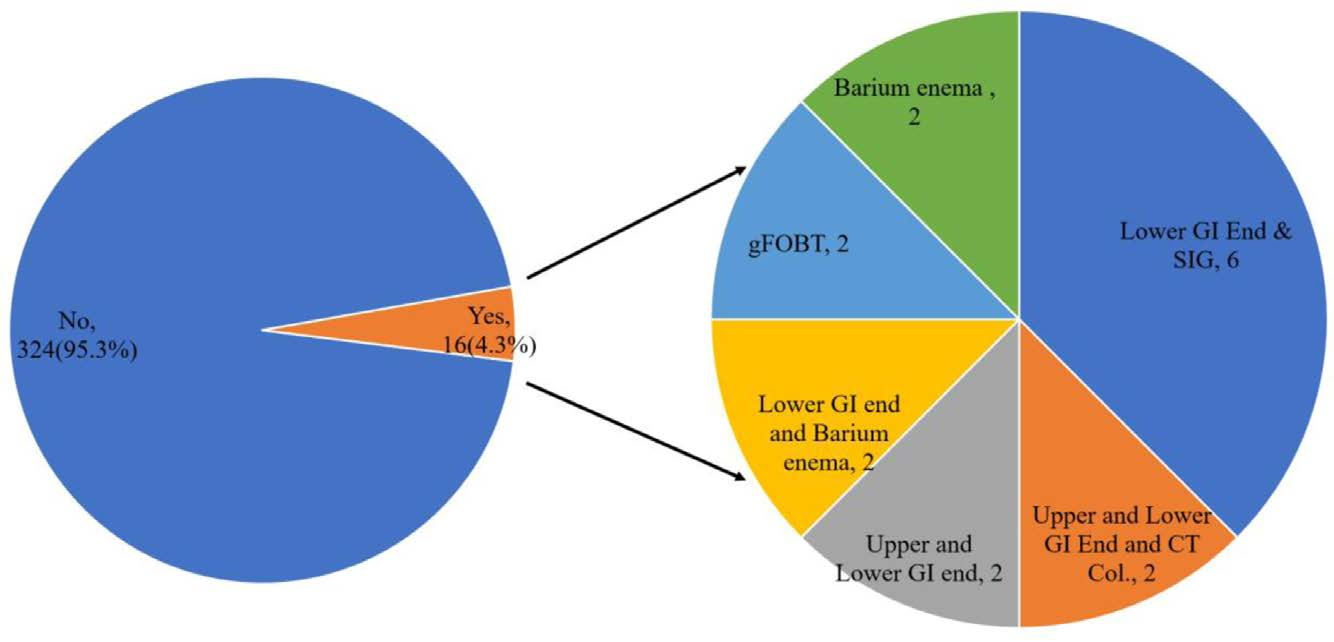
Ever been screened or tested for CRC and types of test done (n = 16). CRC = colorectal cancer.

Figure 1 indicates the responses to the question “Have you ever been screened or tested for CRC?” among a total of 340 respondents. The majority, comprising 95.3% (n = 324), answered in the negative, indicating that they had not undergone CRC screening. Conversely, a smaller proportion, 4.7% (n = 16), responded affirmatively, indicating that they have been screened for CRC. Among, 16 respondents who have been screened or tested for CRC, the most frequently employed screening method is lower GI endoscopy and sigmoidoscopy, accounting for 37.5% (n = 6) of the respondents. Following this, there is an equal distribution of respondents, each constituting 12.5% (n = 2), who underwent upper and lower GI endoscopy with CT colonography, upper and lower gastrointestinal endoscopy, lower GI endoscopy with X-ray colon with barium enema, guaiac-based fecal occult blood test, and X-ray colon with barium enema.

**Figure 2.**
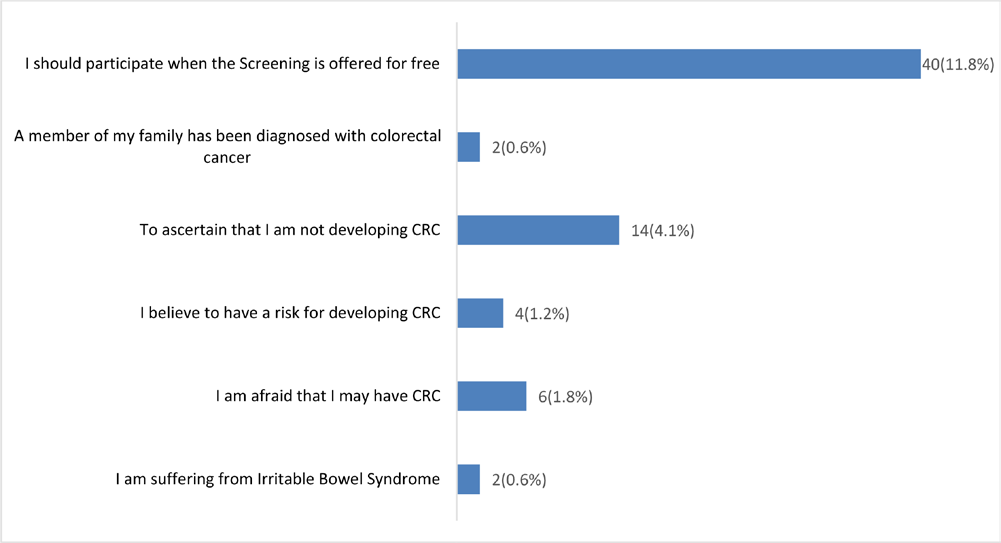
Reasons for participating in CRC screening program (n = 340). CRC = colorectal cancer.

The data on reasons for participating in a CRC screening program among 340 respondents is shown in Figure 2. The majority, constituting 80.0% (n = 272), have never contemplated participating in such a program. Among those expressing an intention to participate, various reasons are identified. A very small percentage of respondents, 0.6% (n = 2) each, state that they intend to participate due to suffering from irritable bowel syndrome or fearing they may have CRC. Another 1.2% (n = 4) express an intention to participate due to a perceived risk of developing CRC, while 4.1% (n = 14) aim to participate to confirm that they are not developing CRC. Similarly, 0.6% (n = 2) of family member’s diagnosis of CRC as their reason to participate. The most common reason, indicated by 11.8% (n = 40) of respondents, is the availability of free screening, suggesting that cost considerations play a significant role in motivating participation.

**Figure 3.**
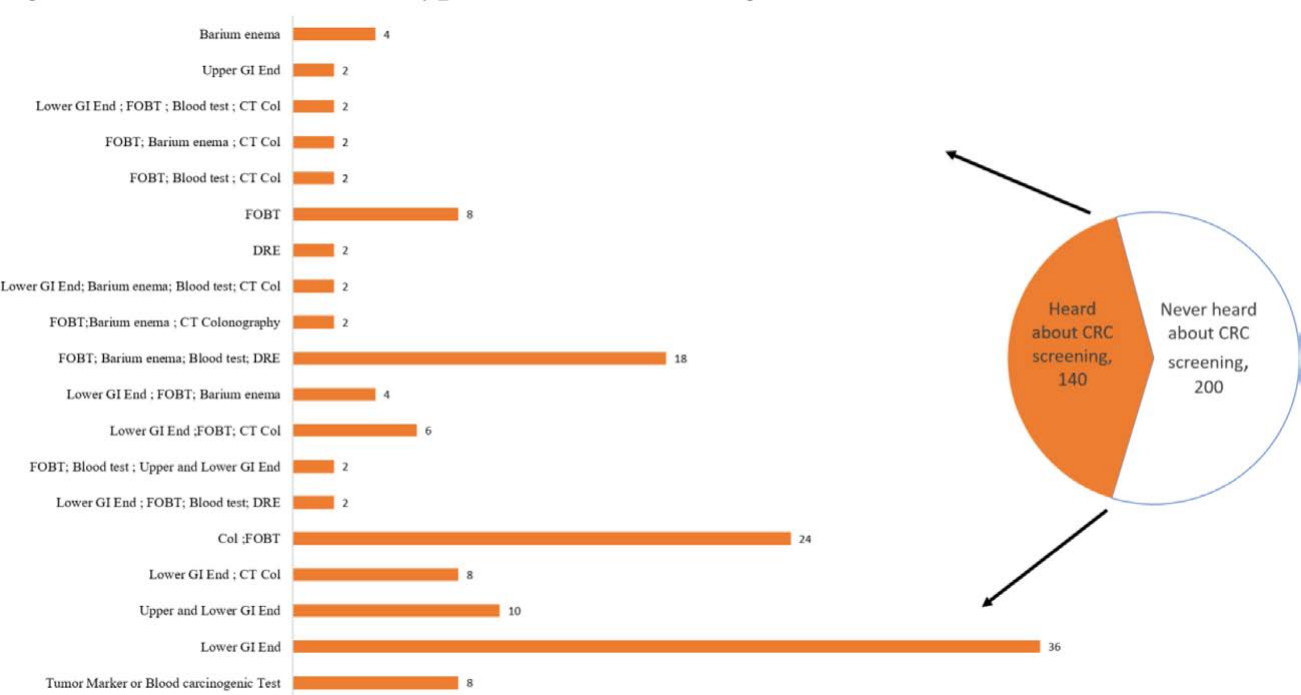
Ever heard about types of CRC screening tests. CRC = colorectal cancer.

Figure 3 shows that, out of the total sample of respondents (N = 340), a significant portion, 58.8% (n = 200), claim to have never heard about CRC screening tests. In contrast, 41.2% (n = 140) of respondents indicate that they have an awareness of these screening tests. Levels of awareness among respondents regarding CRC screening tests. Lower GI endoscopy emerges as the most recognized method, with 36 (25.7%) indicating awareness, followed by colonoscopy with FOBT at 24 (17.1%). While some respondents are familiar with combinations of screening tests, including FOBT with X-ray colon with barium enema and lower GI endoscopy with CT colonography, others show awareness of specific tests such as tumor marker or blood carcinogenic tests. Digital rectal exams and upper GI endoscopy are reported by 2 (1.4%) each.

### 3.3. Perceptions and attitudes

Table 3 provides respondents’ perceptions towards CRC, categorized by different attitudes and the distribution of responses from “strongly agree” to “strongly disagree.” Notably, a majority of respondents express a belief in the preventability of CRC, with 148 strongly agreeing and 152 agreeing to it. In contrast, perceptions about the fatality of CRC vary, with 116 strongly agreeing that it is fatal, while 100 respondents agree, and 92 are neutral. Respondents’ self-assessment of their knowledge of CRC is diverse, with 66 strongly agreeing, 86 agreeing, 88 being neutral, and 52 and 48 disagreeing and strongly disagreeing, respectively. A substantial number, of 194 respondents, strongly agree that the prognosis of CRC improves when discovered early, emphasizing the importance of early detection. Moreover, a majority, of 224 respondents, express a willingness to undergo screening if recommended by a doctor. Additionally, perceptions regarding the risk of CRC in the elderly show varying degrees of agreement, with 126 strongly agreeing, 100 agreeing, 92 being neutral, and 16 and 6 disagreeing and strongly disagreeing, respectively.

**Table 3:** Perceptions and attitude measure towards CRC.

Attitudes	Strongly agree	Agree	Neutral	Disagree	Strongly disagree
CRC (bowel cancer) can be prevented	148	152	36	2	2
I think having colorectal (bowel cancer) is fatal	116	100	92	24	8
I think I have enough knowledge of CRC (bowel cancer)	66	86	88	52	48
The prognosis of CRC is improved when it is discovered in early stages	194	104	34	4	4
If a doctor recommends I to get screened for CRC (bowel cancer) I will do it	224	76	30	4	6
Elderly are at increased risk of CRC (bowel cancer)	126	100	92	16	6

CRC = colorectal cancer.

### 3.4. Perception of study subjects towards CRC

**Figure 4.**
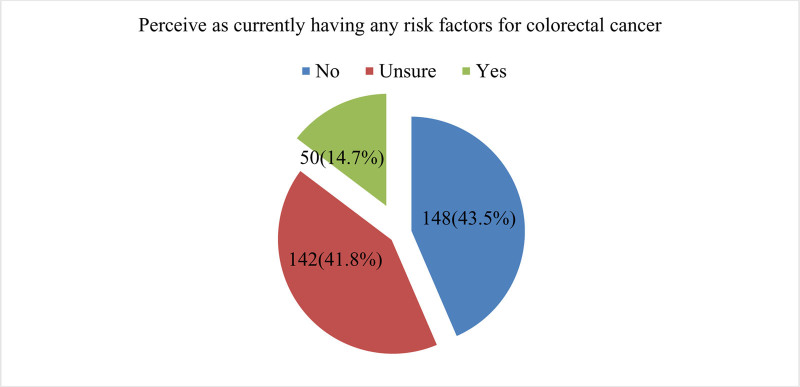
Self-perception of current risk factors for CRC. CRC = colorectal cancer.

Figure 4 presents respondents’ self-perceived risk factors for CRC. The majority, comprising 43.5% (n = 148), perceive themselves as having no risk factors for CRC. A substantial proportion, 41.8% (n = 142), expresses uncertainty about their risk status. On the other hand, 14.7% (n = 50) of respondents believe they currently have risk factors for CRC.

**Figure 5.**
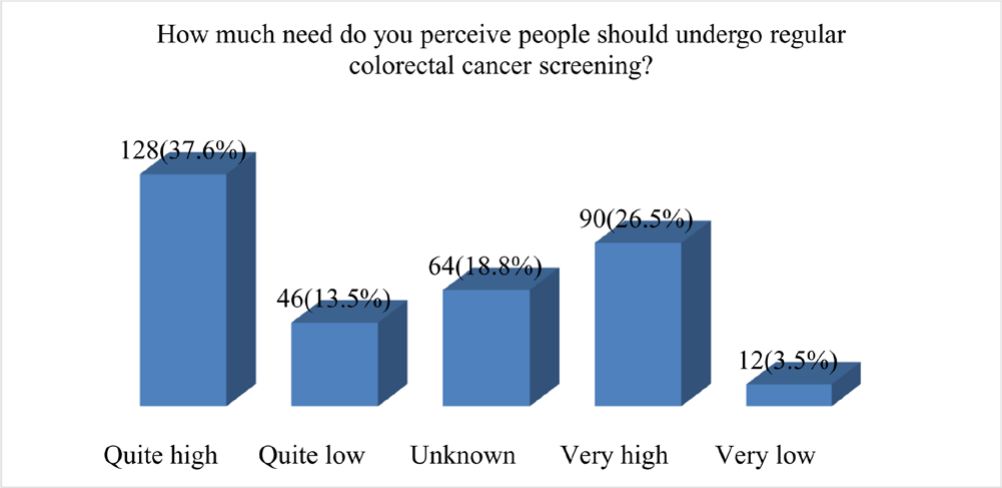
Perceived importance and necessity for regular CRC screening among individuals. CRC = colorectal cancer.

Figure 5 shows that a significant portion, constituting 37.6% (n = 128), believes that the need for regular CRC screening is quite high. In contrast, 13.5% (n = 46) perceive the need as quite low. A notable percentage, 26.5% (n = 90), holds the view that the need for regular screening is very high, indicating a heightened awareness of the importance of CRC prevention. Conversely, a smaller proportion, 3.5% (n = 12), considers the need to be very low. A substantial number of respondents, 18.8% (n = 64), express uncertainty about the perceived need for regular CRC screening.

### 3.5. Information sources and satisfaction

Table 4 explores respondents’ levels of satisfaction regarding the available information concerning CRC screening. The data indicates that 42.4% (n = 144) of the respondents report being somewhat satisfied with the available information. A portion, 32.9% (n = 112), expresses dissatisfaction, while 24.7% (n = 84) report being very satisfied with the information available.

**Table 4:** Satisfaction levels with provided information on CRC screening.

Level of satisfaction	n	%
Somewhat satisfied	144	42.4
Unsatisfied	112	32.9
Very satisfied	84	24.7
Total	340	100.0

CRC = colorectal cancer.

**Figure 6.**
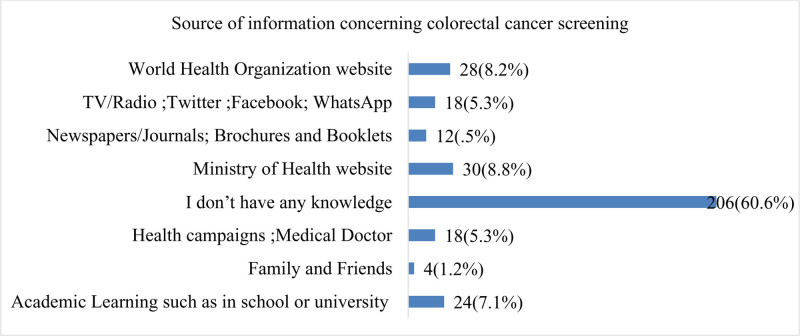
Source of information concerning CRC screening (bowel cancer) (n = 340). CRC = colorectal cancer.

Figure 6 shows that the sources of information concerning CRC screening (bowel cancer) (n = 340). The majority of respondents, comprising 60.59% (n = 206), claim not to have any knowledge about CRC screening. Among those who do know, academic learning from sources such as schools or universities contributes to the awareness of 7.06% (n = 24) of respondents. World Health Organization websites are reported as sources by 8.82% (n = 30) and 8.24% (n = 28) of respondents, respectively. Health campaigns and information from medical doctors are cited by 5.29% (n = 18) of participants. Social media platforms such as TV/Radio, Twitter, Facebook, and WhatsApp collectively contribute to the awareness of another 5.29% (n = 18) of respondents. Family and friends, as well as newspapers/journals, brochures, and booklets, are reported as sources by 1.18% (n = 4) and 3.53% (n = 12) of participants, respectively.

## 4. Discussion

The third most commonly diagnosed cancer is CRC. It is the second most common cause of death among both genders.^[13]^ According to ACS recommendations adults aged 45 years and older with an average risk of CRC should undergo regular screening, all positive results on non-colonoscopy screening test should be further tested by colonoscopy to confirm the diagnosis.^[14]^ Although in our studied population screening rate for CRC was only 4.8% where whereas 3.5% were below 40 years of age. It also shows that the screening rate was higher among male participants (6.3%). In the majority of the studies, CRC screening was conducted between 50 to 64 years and 65 to 75 years of age and there is lower participation in the younger age group.^[15,16]^ According to the results of a systematic review there is also male predominance for testing and diagnosis of CRC, and females are considered as barriers to diagnosis.^[17]^ In some studies it is vice versa where females were more likely to participate in the cancer screening than men.^[18,19]^ The frequency of CRC is rising in younger people, and by 2030, more people aged 20 to 49 are anticipated to have the disease.^[2]^ Therefore, it is important to screen the younger age groups also, as included in this study.

Our studied population showed more screening rates among higher educational status as compared to lower educational status which is also consistent with the findings of other studies as well.^[15,20]^ Employment status was also considered as an influencing factor for the screening of CRC in our population most likely because high cost associated with the screening test which is not afforded by people with unemployment.^[21]^ Marital status is also one of the influencing factors found in our study as a factor influencing CRC screening, which is also suggested from different other studies.^[22,23]^ In our study respondents with a low monthly income showed a relatively higher rate of screening as compared to those with higher income brackets. It might be the reason that in Saudi Arabia health system is completely insured by the Ministry of Health so people do not have to pay from their pocket for the high cost of tests. This finding is not consistent with many other studies which showed a positive association between lower socioeconomic status and lower participation in screening.^[24–26]^

There are many factors associated with CRC, among our participants current smokers exhibit a 5% screening rate and nonsmokers have a 5.1% screening rate whereas according to BMI screening rates for underweight, normal weight, overweight, and obese were 0.0%, 3.1%, 7.1%, and 5.2%, respectively among our participants. Although smoking or tobacco use is a known factor for CRC^[27]^ along with high BMI as well.^[28]^ Therefore we have to encourage people for screening who have high BMI and have a history of smoking. A family history of CRC is one of the strongest factors for acquiring cancer. Those with a family history of CRC have an 11.1% screening rate, whereas those without a family history exhibit a 3.9% screening rate. Participants with first-degree relatives with CRC show a 10.0% screening rate, second-degree relatives exhibit an 11.5% screening rate, and those with no family history have a 3.9% screening rate. However, the rate of screening is higher among those participants who have a strong family history than those who do not have any family history of CRC.^[28]^ Even then people with a strong family history of CRC should be encouraged to undergo regular screening and there should be screening programs supported by health authorities for early detection of cancer.^[29]^ Screening tests can also improve survival and help in decreasing mortality by detecting cancer at an early stage especially when treatment is more effective in eliminating cancer. In our studied population screening rate is very low. The most frequent test done was lower GI endoscopy and sigmoidoscopy 37.5%. A study conducted in the United States also suggested that lower GI colonoscopy is one of the most frequent tests done in different states of the USA.^[30]^ A very small percentage of the participants 0.6% wanted to participate in the screening program because they have irritable bowel syndrome and they have a fear that they might develop CRC, this concept is also evident in another study conducted in Saudi Arabia.^[31]^ About 41.2% of our study participants were aware of different types of CRC screening tests, almost 25.7% were aware of lower GI endoscopy, and 1.4% were aware of digital rectal examination. Whereas a study conducted in Malaysia showed that only 37% of the population was aware of tests for CRC only 5.9% were aware of sigmoidoscopy and 3.2% were aware of fecal occult blood test.^[32]^ A systematic review is also consistent with the findings that low awareness is a causative factor for lower screening rates.^[33]^ Sly JR et al also reported lower awareness as a barrier to the screening of CRC.^[34]^ It is also a factor that along with a lack of information or knowledge and understanding of factual information from the patient, has the potential to incorrectly place a patient in the sporadic CRC screening category instead of the family history category, affecting correct timing of screening and potentially placing the patient at increased risk for the development of CRC.^[35–37]^

The most common source of knowledge about CRC was the internet in a study conducted by Lewandowski et al.^[38]^ Among our participants’ information given by the Ministry of Health was the top source of information and about 24.7% of our participants considered themselves well informed and satisfied about CRC information whereas only 5.3% were considering themselves well aware regarding CRC information in another study conducted in Poland which is quite lower than our study.^[39]^ Markus Dines Knudsen et al reported only 16% highly self-reported awareness level among participants which is quite lower than our participants.^[39]^

Public awareness about family history and genetics as risk factors was higher than in other countries in the region in Kuwait, this might be explained by the fact that the state of Kuwait is a small country as compared to Saudi Arabia where information is easily exchangeable.^[31]^ Around 224 (65.8%) participants think that they will go for a colonoscopy when the doctor advises them. Whereas 55.3% of participants would agree with the colonoscopy suggested by doctors in Poland.^[38]^ Around 194 (57%) of our participants think that CRC can be cured if detected at early stages similarly a study conducted in the Al Baha region of Saudi Arabia showed that around 67.7 % of people believe that CRC can be treated if detected early, this percentage is slightly higher than our studied population.^[40]^ A huge number 80% of our studied participants do not want to undergo screening whereas more than half (58.1%) are ready to get early screening for CRC, even without symptoms.^[40]^ Nearly 116 (34%) believed that CRC is very fatal whereas 25% of Chinese, 22% of Korean, and 61 % of Vietnamese also believe that CRC is fatal although the extent of belief is variable.^[41]^

Around 126 (37%) respondents believed that CRC affects elderly people more although studies also revealed that participation of individuals over 50 in the CRC screening programs ranged from 1.5% to 69%.^[42]^ Around 11% of our participants wanted to participate in the screening program when it was offered free whereas according to the Saudi National Survey willingness to participate is quite high 56% if screening is offered free.^[43]^ Very few 0.6% of participants wanted to undergo screening because of a family history of CRC whereas according to a Saudi national survey, 80% wanted to participate if their family history is positive for CRC, which is also much higher than our studied participants.^[43]^ There were other reasons to participate in screening were people wanted to know that they have cancer or they wanted to know that they are cancer-free. Nearly half 43.5% of our studied population believe that they have risk factors for CRC which is also supported by a meta-analysis that reported a positive association between CRC risk perception and screening behavior.^[44,45]^ Suggesting that the more people believe that they have risk factors more they are inclined towards screening tests. Recent studies in Saudi Arabia have used CRC screening methods like colonoscopy and stool-based tests; however, there is no national CRC screening program in Saudi Arabia, despite it being the one of the most frequent type of cancer among Saudis. The lack of a national screening program is an important issue to be addressed.^[46]^ The ultimate goal is to transform CRC from a life-threatening disease into a manageable condition through widespread, early screening and intervention. A recent study identifies WNT2 as a key promoter of CRC progression through its role in enhancing myeloid-derived suppressor cells accumulation and immunosuppression via the p38 MAPK/Akt pathway. Targeting WNT2 reduces myeloid-derived suppressor cells-mediated immune suppression, suggesting its potential as a biomarker and therapeutic target for CRC.^[47]^

The following factors may have led to certain limitations in the present study. The cross-sectional nature of this study cannot confirm the causality association between the compared variables. The self-reported responses could over or underestimate the results. This study was conducted in Southwestern region of Saudi Arabia and our findings may not present perceptions regarding CRC across Saudi Arabia.

We hope in the future to have all the required resources to do multicentric/nationwide studies.

The low representation of non-Saudi residents (1%) in our study population may be attributed to the convenience sampling technique used to recruit the participants, which may not have captured a representative sample of the expatriate population in the region.

A key limitation of this study is its reliance on convenience sampling, which can lead to selection bias. This method selects readily available participants rather than using random sampling, potentially resulting in a nonrepresentative sample. Consequently, the findings may not be generalizable to the broader population, as certain demographics may be over- or under-represented. To address this limitation, future research should utilize more rigorous sampling techniques, such as stratified random sampling, to ensure a representative sample. To promote diverse representation in our study, we employed several strategies: targeted outreach on various online platforms to engage different demographics, inclusion of demographic questions in the questionnaire for comparative analysis, availability in multiple languages for inclusivity, and prioritization of anonymity and confidentiality to encourage participation. These efforts significantly enhanced the inclusivity of our sample, ensuring a comprehensive representation of diverse perspectives. In conclusion, the implementation of data translation and piloting significantly contributed to reducing bias in our research and implementation processes.

In our study, a higher proportion of participants (60.59%) claimed to have no knowledge about CRC screening. These findings underscore the need for health education and health promotion activities focused on preventing CRC risk factors. Future studies should explore the determinants of CRC and associated factors in younger adults, comparing them to older populations. Additionally, the availability and accessibility of CRC screening, especially in rural areas, need to be strengthened. The following interventions are recommended to improve CRC screening awareness and access:

1. Actionable interventions: implement social media campaigns and tailored outreach programs, which can ultimately lead to greater awareness and higher CRC screening rates.
2. Community health programs: partner with local organizations to raise awareness and provide education to underserved communities.
3. Education and outreach: conduct targeted campaigns, such as community workshops, to inform the public about the importance of screening and procedures, while encouraging rural populations to participate in CRC screening.

## Author contributions

**Conceptualization:** Syed Esam Mahmood, Mousa Ghazwani, Abdullah A. Alsabaani.

**Investigation:** Syed Esam Mahmood, Mousa Ghazwani.

**Formal analysis:** Syed Esam Mahmood, Ausaf Ahmad.

**Methodology:** Syed Esam Mahmood, Mousa Ghazwani, Rishi Kumar Bharti, Razia Aftab Ahmed.

**Project administration:** Syed Esam Mahmood.

**Resources:** Syed Esam Mahmood, Mousa Ghazwani.

**Supervision:** Ayed A. Shati.

**Visualization:** Syed Esam Mahmood, Ausaf Ahmad.

**Writing – original draft:** Syed Esam Mahmood, Fatima Riaz.

**Writing – review & editing:** Syed Esam Mahmood, Abdullah A. Alsabaani.
